# Temporal and spatial trends in insecticide resistance in *Anopheles arabiensis* in Sudan: outcomes from an evaluation of implications of insecticide resistance for malaria vector control

**DOI:** 10.1186/s13071-018-2732-9

**Published:** 2018-03-02

**Authors:** Bashir Adam Ismail, Hmooda Toto Kafy, Jihad Eltaher Sulieman, Krishanthi Subramaniam, Brent Thomas, Abraham Mnzava, Nur Faeza Abu Kassim, Abu Hassan Ahmad, Tessa B. Knox, Immo Kleinschmidt, Martin J. Donnelly

**Affiliations:** 1 Khartoum Malaria Free Initiative, PO Box 1517, Khartoum, Khartoum State Sudan; 2 School of Biological Sciences, Universiti Sains Malaysia, 11800 Minden, Pulau Penang Malaysia; 3 Integrated Vector Management Unit, Federal Ministry of Health, PO Box 303, Khartoum, Sudan; 4 Sennar Malaria Research and Training Centre, Federal Ministry of Health, PO Box 303, Sennar, Sudan; 5 Department of Vector Biology, Liverpool School of Tropical Medicine, Pembroke Place, Liverpool, L3 5QA UK; 6 African Leaders Malaria Alliance (ALMA), 14 Kanisa Road, Corridor Area, P.O. Box 1973, Arusha, Tanzania; 7 Global Malaria Programme, World Health Organization, Geneva, Switzerland; 8 MRC Tropical Epidemiology Group, Department of Infectious Disease Epidemiology, London School of Hygiene and Tropical Medicine, Keppel Street, London, WC1E 7HT UK

**Keywords:** *Anopheles arabiensis*, Deltamethrin, Bendiocarb, Susceptibility bioassay, Combination, Resistance management

## Abstract

**Background:**

Long-lasting insecticidal nets (LLINs) (with pyrethroids) and indoor residual spraying (IRS) are the cornerstones of the Sudanese malaria control program. Insecticide resistance to the principal insecticides in LLINs and IRS is a major concern. This study was designed to monitor insecticide resistance in *Anopheles arabiensis* from 140 clusters in four malaria-endemic areas of Sudan from 2011 to 2014. All clusters received LLINs, while half (*n* = 70), distributed across the four regions, had additional IRS campaigns.

**Methods:**

*Anopheles gambiae* (*s.l*.) mosquitoes were identified to species level using PCR techniques. Standard WHO insecticide susceptibility bioassays were carried out to detect resistance to deltamethrin (0.05%), DDT (4%) and bendiocarb (0.1%). TaqMan assays were performed on random samples of deltamethrin-resistant phenotyped and pyrethrum spray collected individuals to determine *Vgsc*-1014 knockdown resistance mutations.

**Results:**

*Anopheles arabiensis* accounted for 99.9% of any anopheline species collected across all sites. Bioassay screening indicated that mosquitoes remained susceptible to bendiocarb but were resistance to deltamethrin and DDT in all areas. There were significant increases in deltamethrin resistance over the four years, with overall mean percent mortality to deltamethrin declining from 81.0% (95% CI: 77.6–84.3%) in 2011 to 47.7% (95% CI: 43.5–51.8%) in 2014. The rate of increase in phenotypic deltamethrin-resistance was significantly slower in the LLIN + IRS arm than in the LLIN-only arm (Odds ratio 1.34; 95% CI: 1.02–1.77). The frequency of *Vgsc*-1014F mutation varied spatiotemporally with highest frequencies in Galabat (range 0.375–0.616) and New Halfa (range 0.241–0.447). Deltamethrin phenotypic-resistance correlated with *Vgsc-*1014F frequency.

**Conclusion:**

Combining LLIN and IRS, with different classes of insecticide, may delay pyrethroid resistance development, but the speed at which resistance develops may be area-specific. Continued monitoring is vital to ensure optimal management and control.

**Electronic supplementary material:**

The online version of this article (10.1186/s13071-018-2732-9) contains supplementary material, which is available to authorized users.

## Background

The World Malaria Report (2016) estimated that annual malaria incidence in Sudan was 27.4 cases per 1000 population with a case fatality rate of about 2 deaths per 100,000 [1]. More than 96% of malaria cases are due to *Plasmodium falciparum* (Sudan-Malaria Indicator Survey 2012, unpublished data). *Anopheles arabiensis* is the principal malaria vector in all parts of the country [2, 3], with *Anopheles gambiae* (*s.s.*) and *Anopheles funestus* having a minor, focal role in malaria transmission in southern and eastern parts of the country [4, 5]. Sudan has a long history of malaria vector control, but efforts have been limited and ultimately unsustained. Between 1900 and 1950, malaria transmission was successfully suppressed in urban settings through larval control activities which included the use of Paris Green, diesel oil, larvivorous fish and environmental water management [6, 7].

With the advent of the malaria eradication era in the 1950s, indoor residual spraying (IRS) with BHC (Benzene hexachloride) was initiated as the main measure against adult vectors in malarious areas such as Gezira and Khashm Elgirba irrigated schemes [7, 8]. In 1965, BHC was replaced by DDT (dichloro-diphenyl-trichloroethane), as the insecticide of choice in Sennar and Gezira provinces [7]. Unfortunately, the campaign was terminated by 1970 due to a number of factors including insecticide resistance. In response to the resistance problem, an organophosphate insecticide, malathion, was introduced for IRS in 1975 in Gezira irrigated areas. Malathion remained in use until late 1979, when it was replaced by fenitrothion [7]. Also in 1975, an organophosphate insecticide, temephos, was introduced for larval control. During the 1990s, several synthetic pyrethroid insecticides were introduced for public health use. IRS spraying of deltamethrin became the most common practice with permethrin used for ultralow volume (ULV) and thermal-fog space spraying to control nuisance mosquitoes (Sudan-IVM: Integrated Vector Management Strategic Plan 2014–2018, unpublished data). Pyrethroid-treated bed net coverage has also increased markedly in recent years [9].

In 2013, 5.9 million deltamethrin-treated, long-lasting insecticidal mosquito nets (LLINs; PermaNet 2.0) were distributed in 13 states, bringing the total LLINs distributed during 2010–2015 to approximately 13 million with an estimated coverage of 69% of the target rural population. The distributions are implemented through campaigns using communication for behavioural impact (COMBI) methodology, with all nets provided by the Global Fund (Sudan-IVM: Integrated Vector Management Strategic Plan 2014–2018, unpublished data). In addition to LLIN scale-up and in an attempt to retard the emergence of pyrethroid resistance in *An. arabiensis*, bendiocarb (Ficam®) was introduced for IRS application in Gezira irrigated area. Bendiocarb IRS is being extended to cover all areas where pyrethroid resistance has been reported (Sudan-IVM strategic plan 2014–2018). LLINs and IRS are well proven interventions for malaria control [10, 11] so the development and spread of insecticide resistance to all four public health insecticides currently recommended by WHO is a major concern [12, 13].

Insecticide resistance is widespread in the main malaria vector *An. arabiensis* in Sudan.

Historically, *An. arabiensis* was resistant to BHC (first report 1964) and DDT (first report 1970) in a sugar cane production area in Gezira State [14]. Whilst, in 1979, malathion resistance was reported in the same state [15, 16]. More recently in the 2000s, *An. arabiensis* populations from Khartoum State showed evidence of resistance to DDT, permethrin and malathion, but not to deltamethrin, lambdacyhalothrin, carbamates and fenitrothion [17, 18]. Additional observations of resistance of *An. arabiensis* to malathion, DDT, permethrin and lambdacyhalothrin insecticides were reported from a variety of locations in central and eastern parts of the country [2, 19–22].

Mechanistically, insecticide resistance commonly occurs due to alterations in the target site of the active ingredient and/or enhanced detoxification or sequestration. For pyrethroids and DDT target site insensitivity results from mutations in the Voltage gated sodium channel (*Vgsc*) which is the target site of these insecticides. A well-described resistance mutation, *Vgsc-*1014F, is widely reported in Gezira, Sennar, Kassala States [2, 21, 23], Khartoum, While Nile and Blue Nile State [22]. Target site insensitivity to carbamates and organophosphates occurs in the acetyl-cholinesterase (*AChE*) locus [24]; recently this mutation has been observed in populations from Khartoum but at a very low frequency level [25]. Metabolism-based resistance was investigated in malathion resistant *An. arabiensis* from Gezira and it was concluded that a carboxylesterase enzyme was the basis of the resistance [15]. Recently, a microarray analysis of a permethrin-resistant population of *An. arabiensis* from Wad Medani showed over-expression of two cytochrome P450s; *Cyp6m2* and *Cyp6p3* were associated with the resistance phenotype [23]. In 2010, before starting the implementation of the Sudan Insecticide Resistance and Control (SIRAC) project, a comprehensive malaria indicator survey (MIS) was carried out in all 140 clusters (i.e. villages) with an emphasis on calculating parasitaemia prevalence, coverage of IRS and LLINs as well as collecting population and household census data. The SIRAC project also included monitoring of insecticide resistance mutations in the *Vgsc* and in the acetyl-cholinesterase (*ACHE*) locus in all 140 clusters. This work is part of a larger cluster randomized trial designed to: (i) Compare the impact of LLINs *vs* LLINs + IRS on malaria epidemiological indices (*P. falciparum* prevalence and malaria case incidence), (ii) Estimate the impact of pyrethroid resistance on malaria epidemiological indices, and (iii) Determine if a combination of LLINs (deltamethrin) and IRS (with either deltamethrin or bendiocarb) acted synergistically to delay the emergence of insecticide resistance.

Using the cluster specific results of the 2010 baseline survey, restricted randomisation was carried out to allocate 70 of the 140 clusters to receive IRS in addition to LLINs, thereby ensuring balance between study arms [26]. More details of the study design may be found in Kleinschmidt et al. [27] and Kafy et al. [28]. In this manuscript we present results describing the patterns of insecticide resistance and the impact of combined intervention on the evolution of resistance.

## Methods

**Fig. 1:**
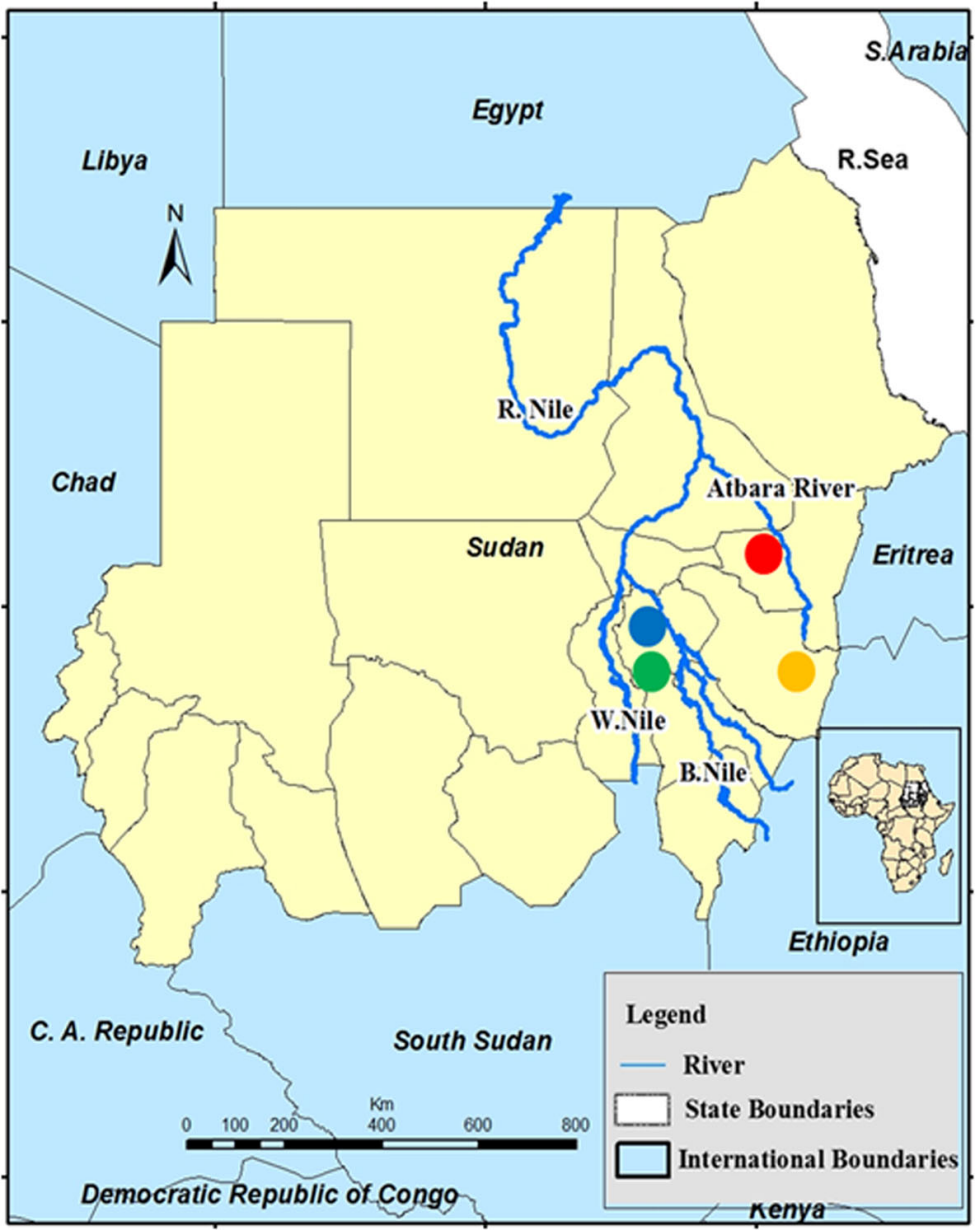
Sudan map showing the location of the study areas: El Hoosh (blue circle), Hag Abdalla (green), Galabat (yellow) and New Halfa (red)

The project was conducted in four malaria-endemic districts of Sudan: El Hoosh, Hag Abdalla, Galabat and New Halfa (Fig. 1).

**Fig. 2:**
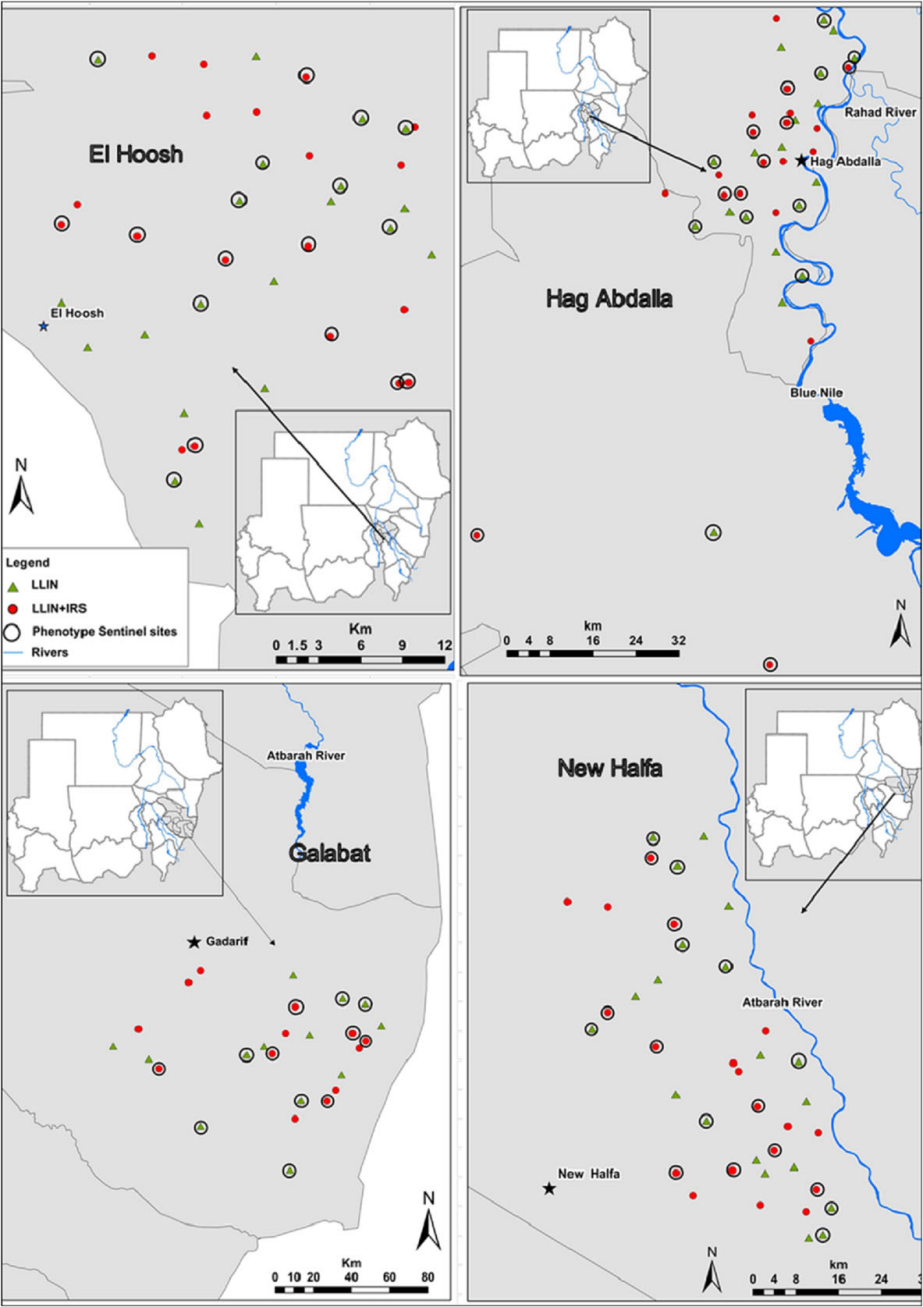
Map showing the distribution of 66/140 clusters in El Hoosh, Hag Abdalla, Galabat and New Halfa used for insecticide resistance monitoring 2011–2014

El Hoosh is in the southwest of Gezira State, approximately 45 km from Wad-Medani, the state capital. The area comprises 28,685 houses grouped in 176 villages with a total of 138,253 inhabitants with > 80% of households reliant upon farming as a primary income source. Thirty eight villages were randomly allocated to LLIN (deltamethrin; *n* = 19 clusters) or LLIN + IRS (bendiocarb; *n* = 19 clusters) intervention arms (Fig. 2).

Hag Abdullah is located on the western bank of the Blue Nile River, approximately 20 km southeast of El Hoosh. There are 101,923 inhabitants, in 21,253 houses in 107 villages with the majority of villagers being farmers. Thirty eight villages were selected and randomly allocated to LLIN (deltamethrin; *n* = 19 clusters) or LLIN + IRS (bendiocarb; *n* = 19 clusters) intervention arms (Fig. 2). Both El Hoosh and Hag Abdalla are irrigated by a canal system originating at the Sennar Dam on the Blue Nile River. Cotton, wheat, groundnuts, sorghum and vegetables are cultivated with agricultural activities running from July to October. The climate is hot and dry in summer (March to June) the average daily temperature is 32 °C and relative humidity 20%. During the cooler, dry winter (October to February), the average daily temperature is 22 °C, and relative humidity 30%. Average annual rainfall is 225 mm per annum. *An. arabiensis*, the primary vector of malaria, has developed resistance to DDT, deltamethrin, permethrin and malathion [2].

Galabat is located in Gedarif State approximately 80 km from Gedarif town and bordering Ethiopia. The area comprises 118,854 household in 197 villages with total of 599,270 inhabitants. The population is predominantly dependent on rain-fed agriculture. Climatically, the area is within the dry savannah region, with a short rainy season (June to September), and long dry season (October to May). Annual rainfall ranges between 700–1200 mm. Annual average daily temperatures range between 31 °C and 44 °C with April and May being the hottest months of the year. Malaria transmission is seasonal from July to October. *Anopheles arabiensis* is the main vector of malaria in the area, with *An. funestus* implicated as having a minor role in malaria transmission [29]. There are no reports of insecticide resistance, and LLINs are the main vector control method. In this study area 26 villages were selected and randomly allocated to LLIN (deltamethrin; *n* = 13 clusters) or LLIN + IRS (*n* = 13 clusters) intervention arms. For the LLIN + IRS arm the chemical used for IRS was deltamethrin for in 2011 and 2012 and then bendiocarb from 2013 onwards (Fig. 2).

New Halfa is located in the semi-arid belt of Sudan approximately 500 km east of Khartoum within the New Halfa sugar cane and New Halfa irrigation scheme in Kassala State. The area has a population of 241,402 living in 35,940 houses scattered among 107 villages. Most villages are situated along the Atbara River, where cotton, sugar, wheat, sorghum and a variety of vegetables are cultivated. Climatically, the area is dry savannah with rainfall ranging between 300 and 411 mm per annum. The rainy season lasts from July to early October. Temperatures range between 16 °C and 45 °C. The principal malaria vector species *An. arabiensis* has shown resistance to DDT, deltamethrin and malathion [19, 21]. Indoor residual spraying with deltamethrin during the rainy season was formerly the main strategy for malaria vector control. The number of clusters is same as in El Hoosh and Hag Addalla, with 19 clusters in both the LLIN and LLIN + IRS arms (Fig. 2).

### Mosquito sampling and rearing

To monitor insecticide resistance, nine sentinel clusters were randomly selected from each of the two study arms in El Hoosh, Hag Abdalla and New Halfa, whilst in Galabat six sentinel clusters per study arm were randomly selected resulting in a total of 66. In each year during the late rainy season in September-December, *Anopheles* larvae and pupae were collected from a range of local breeding habitats from the sentinel clusters and transported to field entomology labs at Sennar Malaria Research and Training Centre; Gedarif or New Halfa. In the laboratory, samples were sorted into instars and placed in separate plastic rearing containers. Larvae were fed on finely ground Tetramin fish food (Aquafin cichlid®, Bangalore, India) and reared to adulthood. Emerging adults were transferred into cages and maintained at 26 ± 1°C, 70–80% relative humidity and provided with 10% sugar solution until used for insecticide bioassay tests.

### Insecticide susceptibility test

Bioassays were performed on morphologically identified *An. gambiae* complex mosquitoes using the standard WHO susceptibility test kit with discriminating concentrations of (0.05%) deltamethrin (4%) DDT and (0.1%) bendiocarb [30, 31]. The impregnated and control papers were supplied by Vector Control Research Centre-Universiti Sains Malaysia (VCRC-USM) and were used up to six times. Two to three day-old, sugar-fed adult female mosquitoes were tested. Sets of four replicate tubes with 20–25 adult mosquitoes per tube were tested with each insecticide and control (impregnated with acetone and silicone oil used as diluents). After 60 min exposure, mosquitoes were transferred into holding tubes and provided with cotton wool soaked with a 10% sucrose solution. Mortality was calculated after a 24 h holding period. Dead and surviving mosquitoes from each bioassay were kept separately in Eppendorf tubes over silica gel for subsequent molecular analysis.

### Pyrethrum spray collection

In October 2010, an extensive pre-intervention entomological survey was preformed to collect indoor resting mosquitoes using pyrethrum spray collection (PSC) and aspirators. In total, 7800 *Anopheles gambiae* complex mosquitoes in 140 clusters across the four areas were collected and PCR assayed for species identification and *Vgsc*-1014 genotype. Following the first implementation year (2011) when *Vgsc*-1014 genotyping was conducted in only 56 out of the 66 sentinel clusters it was decided that *kdr* genotyping would be conducted in all 140 clusters as a proxy for phenotypic resistance. From 2012, during the main transmission season (September-November), PSCs were used to sample indoor-resting mosquitoes from 3 randomly selected houses in 74 clusters (phenotyping collections were conducted in the remaining 66 sentinel sites). Collections were conducted between 07:00 and 10:00 h. In each room, the floor and furniture were covered by white sheets (size 4 × 4 m), and windows/eaves were carefully closed. One collector inside the house sprayed the roof and walls with a pyrethroid / aerosol (FLYTEX TM containing; Tetramethrin 0.2%, Cyluthrin 0.025% and PBO 1.0%, Laboratoire Elie for Insecticides, Khartoum Sudan). Then the house was left closed for 10–15 min, after which dead mosquitoes were collected from the sheets, sorted to genus level, and transferred to the field laboratory on moist filter papers in Petri dishes. In the laboratory, only *Anopheles* mosquitoes were identified morphologically to species level [32, 33]. All mosquitoes that were morphologically identified as belonging to the *Anopheles gambiae* complex were preserved in Eppendorf tubes on silica gel for PCR-based species identification and *Vgsc*-1014 genotyping. A subset of 24 individuals selected at random from the deltamethrin phenotyped specimens (sentinel sites) or the same number from the PSC samples were selected for *Vgsc*-1014F screening. Given the predominance of *An. arabiensis* from 2012, approximately 15% of genotyped specimens (4/24 per cluster) from all 140 were identified to species. This assumed that all were found to be *An. arabiensis* if this was not the case additional screening would be required, this was not necessary.

Genomic DNA extraction was performed following Livak et al. [34]. *Anopheles gambiae* complex mosquitoes were identified to specific status using the standard ribosomal DNA PCR [35]. The *Vgsc*-1014 and Ace1-119 (*AChE*) mutations were screened using TaqMan assays [36, 37] at the Sennar Malaria Research and Training Centre.

### Data analysis

Analysis of variance (ANOVA) was performed on arcsine transformed data to compare the differences in overall mean mortality and *Vgsc*-1014F allele frequencies between areas and years. The genotypic frequencies for each area over year were compared to Hardy-Weinberg expectations using exact test procedures. The emergence of deltamethrin resistance was investigated using resgression analysis to correltate percent mortality data for each pair of consecutive years, and any positive trend (i.e. slope values) suggesting resistance increase. To assess the potential impact of intervention arm on pyrethroid resistance development we used a generalized linear mixed effects model (GLMM) implemented in R and using the *lme4* library [38]. Analyses were carried out on 30 sentinel sites for which deltamethrin bioassay mortality data were available for all four years. Sentinel sites from Galabat were excluded as there was a change in the active ingredient used in the IRS campaign from deltamethrin (years 2011 and 2012) to bendiocarb (years 2013 and 2014). Fixed effects were year, intervention arm and area; random effect was cluster. A binomial error distribution and a logistic link function was used. Significance was assigned though comparison of likelihood ratio tests of the final full model which included intervention arm against the model without this variable. A similar analysis was performed on the *Vgsc*-1014F frequency data. Analysis was conducted on frequency data from clusters which had complete frequency data from the years after the mass LLIN distribution (2011–2014) and from all areas except Galabat (no clusters = 44). Likewise, the correlation between deltamethrin phenotype mortality and *Vgsc*-1014F was analysed using GLMM with area and year as fixed effects. All statistical analyses, except where mentioned, were performed using STATA version 12.00, or the significance level was set at α = 0.05.

## Results

### Species identification

During 2010–2014, a total of 2580 samples were assayed by PCR; 93.5% (*n* = 2411) specimens were successfully amplified of which 99.9% were *An. arabiensis*. Two specimens of *An. gambiae* (*s.s.*) were identified from the Hag Abdalla area. Henceforth all analyses are presumed to be conducted on *An. arabiensis*.

### Resistance phenotyping

Over four years, a total of 74,024 F_0_ female *Anopheles* (raised from field-collected larvae) were tested for susceptibility to deltamethrin (0.05%), DDT (4%) or bendiocarb (0.1%); of these 50,994 (69%) were exposed to insecticide impregnated papers and 23,030 (31%) were used as control. In total 31,182 adult female (*n*_exposed_ = 21,827; *n*_control_ = 9355) were bioassayed against deltamethrin, 19,153 (*n*_exposed_ = 13,038; *n*_control_ = 6115) against DDT and 23,689 (*n*_exposed_ = 16,129; *n*_control_ = 7560) against bendiocarb.

**Fig. 3:**
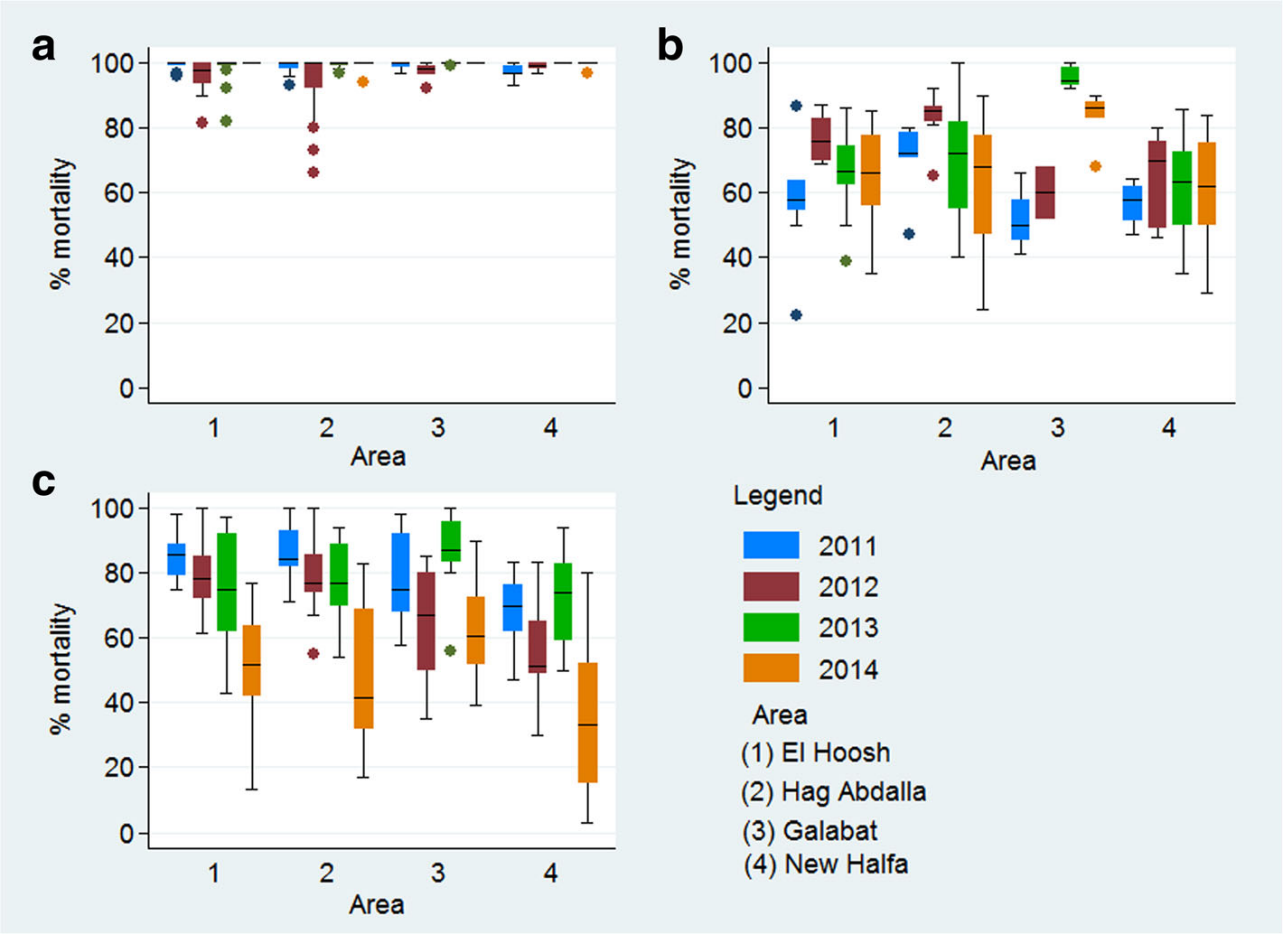
Twenty-four hours % mortality of *An. arabiensis* mosquitoes exposed for 1 h to WHO papers impregnated with **a** bendiocarb, **b** DDT and **c** deltamethrin in Sudan 2011–2014

*Anopheles arabiensis* populations from all sites were susceptible to bendiocarb; overall mean mortality rates ranged between 97.8–100%. A slight trend of reduced susceptibility in 2011 and 2012 was reversed during 2013–2014 (Fig. 3a, Additional file 1; Table S1). All populations were resistant to DDT and deltamethrin. Against DDT, there were significant differences (*F*_(3, 137)_ = 4.62, *P <* 0.004) in resistance level between the four areas, and years (*F*_(3, 137)_ = 6.07, *P <* 0.001), the highest resistance observed in populations from New Halfa where mortality ranged between 58–70% (*P* < 0.005) (Fig. 3b, Additional file 2; Table S2). Deltamethrin resistance levels also varied significantly between study areas (*F*_(3, 213)_ = 13.22, *P* < 0.001), with a marked increase in resistance over the four years (*F*_(3, 213)_ = 53.29, *P* < 0.001). The highest resistance frequency was found in populations from New Halfa with mortality rates ranged from 33 to 74% during the four year monitoring period (Fig. 3c, Additional file 3; Table S3).

### Cluster-specific inter-year correlation

**Fig. 4:**
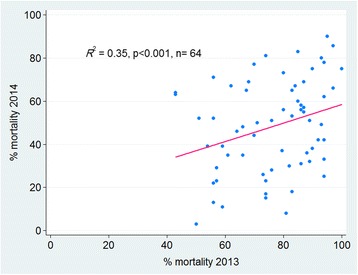
Correlation between cluster-specific deltamethrin susceptibility (% mortality) across all four study areas in Sudan

There was an increase in deltamethrin resistance between each pairs of consecutive years. The percentage loss of susceptibility per year was 10.3% (95% CI: 5.5–15.0%, *P* < 0.0001) in 2011 *vs* 2012 (effect size = 0.13), whilst 2013 *vs* 2014 the loss per year was 29.1% (95% CI: 23.2–34.8%, *P* < 0.0001, effect size = 0.35). However, there was no significant correlation in bioassay percent mortality between 2012 and 2013 (*P* > 0.05) (Fig. 4).

### Resistance genotyping

*Vgsc*-1014F and *Vgsc-*1014S screening was conducted in all study clusters from 2010 to 2014 with the exception of 2011 when it only performed on clusters where deltamethrin phenotyping was conducted. Of a total of 17,231 *An. arabiensis* (*n*_2010_ = 3494; *n*_2011_= 3657; *n*_2012–2014_ = 3360/year) selected for analysis genotyping was successfully completed on 98% of specimens (Table 1).

**Table 1:** Genotypic frequency in *An. arabiensis* populations from the four areas in Sudan 2010–2014. A *P*-value < 0.05 indicates significant departure from HWE

Area	Year	Sample size	Scored	Failed	Genotype frequency (%)			HWE-test
					LL	LF	FF	
El Hoosh	2010	950	834	116	519 (62.2)	288 (34.5)	27 (3.3)	*χ*^2^_(1)_ = 2.93; *P* > 0.05
	2011	1082	1072	10	364 (34)	561 (52.3)	147 (13.7)	*χ*^2^_(1)_ = 8.94; *P* < 0.001
	2012	912	879	33	501 (57)	327 (37.2)	51 (5.8)	*χ*^2^_(1)_ = 0.06; *P* > 0.05
	2013	912	900	12	561 (62.3)	302 (33.6)	37 (4.1)	*χ*^2^_(1)_ = 0.21; *P* > 0.05
	2014	912	912	0	608 (66.7)	269 (29.5)	35 (3.8)	*χ*^2^_(1)_ = 0.58; *P* > 0.05
Hag Abdalla	2010	950	906	44	541 (59.7)	317 (35)	48 (5.3)	*χ*^2^_(1)_ = 0.03; *P* > 0.05
	2011	975	973	2	364 (37.4)	518 (53.2)	91 (9.4)	*χ*^2^_(1)_ = 23.59; *P* < 0.0001
	2012	912	883	29	458 (51.9)	371 (42)	54 (6.1)	*χ*^2^_(1)_ = 3.48 *P* > 0.05
	2013	912	903	9	555 (61.5)	298 (33)	50 (5.5)	*χ*^2^_(1)_ = 1.41 *P* > 0.05
	2014	912	909	3	625 (68.8)	258 (28.3)	26 (2.9)	*χ*^2^_(1)_ = 0.01; *P* > 0.05
Galabat	2010	644	626	18	191 (30.5)	248 (39.6)	187 (29.9)	*χ*^2^_(1)_ = 26.98; *P* < 0.0001
	2011	853	848	5	65 (7.7)	431 (50.8)	352 (41.5)	*χ*^2^_(1)_ = 18.57; *P* < 0.0001
	2012	624	608	16	116 (19.1)	249 (40.9)	243 (40)	*χ*^2^_(1)_ = 12.52; *P* < 0.001
	2013	624	618	6	151 (24.4)	316 (51.2)	151 (24.4)	*χ*^2^_(1)_ = 0.31; *P* > 0.05
	2014	624	621	3	234 (37.7)	305 (49.1)	82 (13.2)	*χ*^2^_(1)_ = 1.25; *P* > 0.05
New Halfa	2010	950	921	29	332 (36)	443 (48.1)	146 (15.9)	*χ*^2^_(1)_ = 0.01; *P* > 0.05
	2011	747	745	2	138 (18.5)	391 (52.5)	216 (29)	*χ*^2^_(1)_ = 2.79; *P* > 0.05
	2012	912	912	0	272 (29.8)	438 (48)	202 (22.1)	*χ*^2^_(1)_ = 1.04; *P* > 0.05
	2013	912	909	3	368 (40.5)	431 (47.4)	110 (12.1)	*χ*^2^_(1)_ = 0.89; *P* > 0.05
	2014	912	912	0	413 (45.3)	454 (49.8)	45 (4.9)	*χ*^2^_(1)_ = 32.66; *P* < 0.0001

*Abbreviations*: *LL* homozygous susceptible; *LF* heterozygous; *FF* homozygous resistant; *HWE* Hardy-Weinberg expectation

In El Hoosh and Hag Abdalla, the samples in 2011 showed a significant departure from the Hardy-Weinberg expectation (HWE) due to an excess of heterozygotes (El Hoosh: percentage = 52.3%, *χ*^2^ = 8.94, *df* = 1, *P* < 0.001; Hag Abdalla: percentage = 53.2%, *χ*^2^ = 23.59, *df* = 1, *P* < 0.0001). A significant deviation from HWE was observed in Galabat during 2010, 2011 and 2012 (all *P* **<** 0.001) and in New Halfa in 2014 (*χ*^2^ = 32.66, *df* = 1, *P* < 0.0001) (Table 1).

**Fig. 5:**
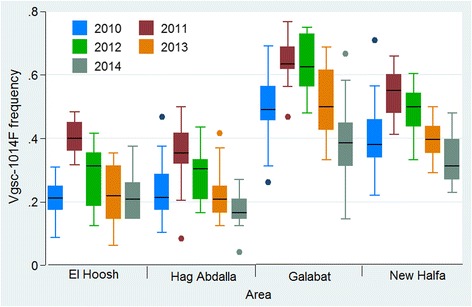
*Vgsc*-1014F frequencies in *An. arabiensis* populations from the four study areas in Sudan 2010–2014

In 2010, *Vgsc-*1014S was detected in three areas at very low frequency (0.001 to 0.0087), whilst, *Vgsc-*1014F was observed in all samples screened from 140 clusters across the four study areas. During 2011–2014, the *Vgsc-*1014S mutation was not detected in the study populations so only *Vgsc-*1014F data are reported by study area and year. There were marked differences in *Vgsc-*1014F frequency between areas (*F*_(3, 608)_ = 239.1, *P* < 0.001). The *Vgsc-*1014F frequencies were consistently high in Galabat (frequency range 0.375–0.616) followed by New Halfa (range 0.241–0.447) (Fig. 5).

In all areas there was significant decrease in *Vgsc*-1014F frequency over years (*F*_(4, 606)_ = 16.7, *P* < 0.001), and the lowest *Vgsc-*1014F frequencies in all areas were observed in 2014 (Fig. 5). No acetyl-cholinesterase (*AChE*) resistance mutation was detected in any samples genotyped at baseline (*n* = 2653) or specimens surviving (*n* = 206) bendiocarb WHO susceptibility bioassays.

### Impact of combined interventions on resistance evolution

**Fig. 6:**
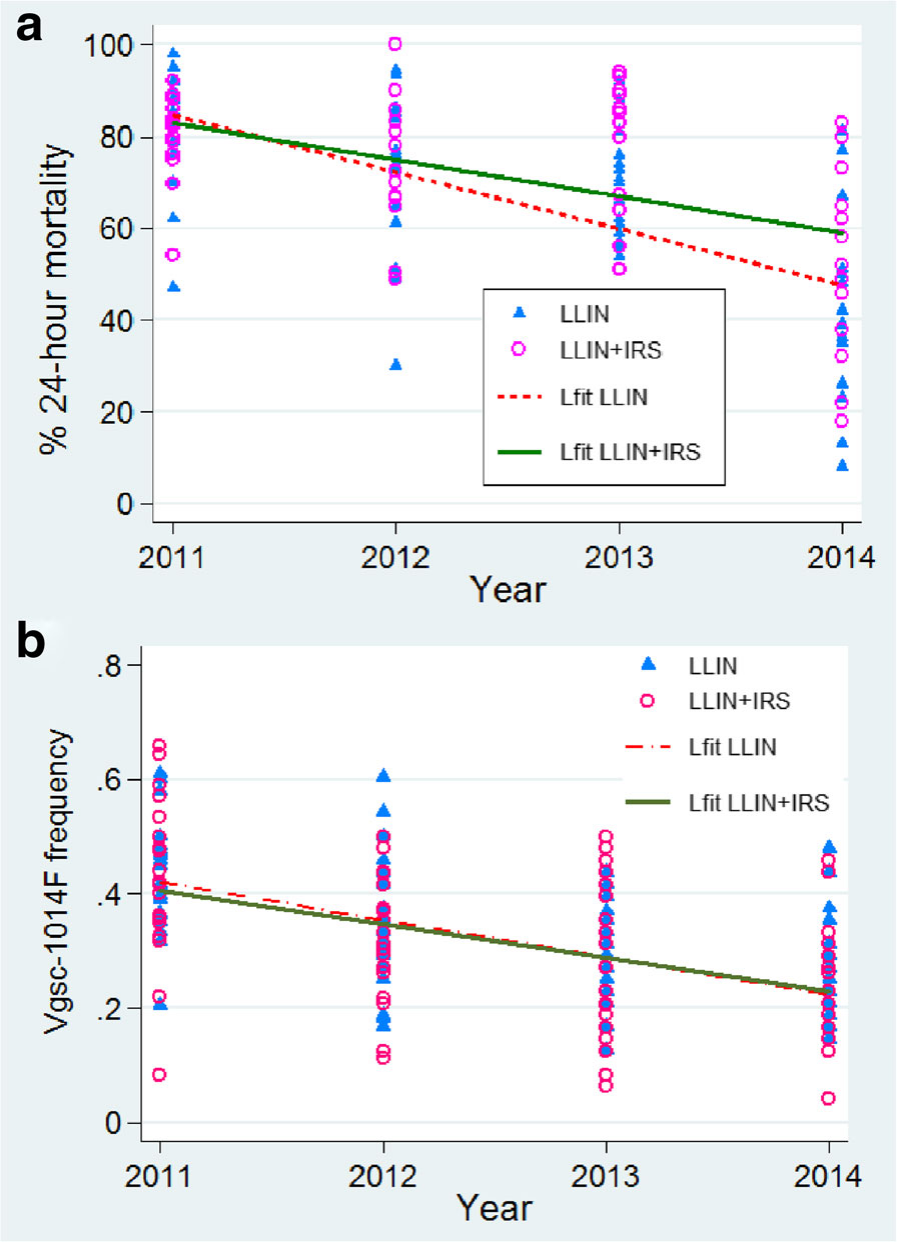
Impact of combine intervention of LLIN + IRS (with bendiocarb) in pyrethroid resistance *An. arabiensis*, from Sudan 2011–2014. **a** Deltamethrin bioassay percent mortality, **b** *Vgsc*-1014F frequency. Triangles denote cluster-specific mortality or Vgsc-1014F in LLIN arm, and open circles denote clusters sme variables in LLIN + IRS arm. The fit line for the scatterplot (**a**) is less steep in LLIN + IRS than in LLIN arm, suggesting delay in deltamethrin resistance development

WHO susceptibility bioassays were conducted in 49 out of the 66 sentinel sites in 2011, 50 in 2012 and 65 sites in 2013 and 2014, whilst *Vgsc-*1014F screening was performed for all 140 clusters in 2010, 2012, 2013 and 2014 but in 56 clusters only in 2011. The generalized linear mixed effects models (GLMM), showed that while resistance increased over the course of the study the rate of increase was lower in the dual intervention arm (Odds ratio 1.34; 95% CI: 1.02–1.77; test of improved model fit with arm *P* < 0.05). This suggests that the use of dual insecticide interventions which combine different classes of insecticide can delay resistance development (Table 2, Fig. 6a).

**Table 2:** Deltamethrin susceptibility % mortality of *An. arabiensis* populations from El Hoosh, Hag Abdalla and New Halfa study areas per intervention arms 2011–2014

Year	Area by intervention arm^a^							
	El Hoosh		Hag Abdalla		New Halfa		Total	
	LLIN (*n* = 6)	LLIN + IRS (*n* = 5)	LLIN (*n* = 6)	LLIN + IRS (*n* = 5)	LLIN (*n* = 4)	LLIN + IRS (*n* = 4)	LLIN (*n* = 16)	LLIN + IRS (*n* = 14)
2011	86.1	84.2	88.9	83.9	63.8	70.0	81.6	80.0
2012	80.6	74.8	79.0	83.1	48.8	62.0	72.0	74.1
2013	71.7	72.3	71.0	87.6	64.5	72.0	69.6	77.7
2014	45.5	54.0	46.7	57.6	26.3	43.0	41.1	52.1

^a^Galabat was excluded due to change in insecticide used for IRS (deltamethrin in 2011/2012 and bendiocarb in 2013/2014)

*Abbreviation*: n, number of sentinel clusters for which data are available for all four years

The GLMM analyses of *Vgsc-*1014F frequencies were conducted on data from the 44 clusters for which there was *kdr* genotypic data for all four years post LLIN distribution (2011–2014) (Table 3). Whilst there was evidence of decreasing *Vgsc***-**1014F frequency (i.e. increase in susceptible alleles) over time in both intervention arms there was no significant difference in the rate of change of frequency (Odds ratio 1.008; 95% CI: 0.91–1.11; test of improved model fit with arm *P* > 0.05) (Fig. 6b).

**Table 3:** *Vgsc*-1014F frequency in *An. arabiensis* populations from El Hoosh, Hag Abdalla and New Halfa study areas per intervention arms 2011–2014

Year	Area by intervention arm^a^							
	El Hoosh		Hag Abdalla		New Halfa		Total	
	LLIN (*n* = 7)	LLIN + IRS (*n* = 7)	LLIN (*n* = 8)	LLIN + IRS (*n* = 8)	LLIN (*n* = 7)	LLIN + IRS (*n* = 7)	LLIN (*n* = 22)	LLIN + IRS (*n* = 22)
2011	0.425	0.38	0.356	0.341	0.521	0.568	0.339	0.334
2012	0.298	0.255	0.278	0.309	0.488	0.414	0.351	0.325
2013	0.211	0.179	0.228	0.221	0.366	0.435	0.267	0.275
2014	0.229	0.226	0.159	0.181	0.342	0.342	0.240	0.247

^a^Galabat was excluded due to change in insecticide used for IRS (deltamethrin in 2011/2012 and bendiocarb in 2013/2014)

*Abbreviation*: n, number of sentinel clusters for which data are available for all four years

### Genotype phenotype associations

**Fig. 7:**
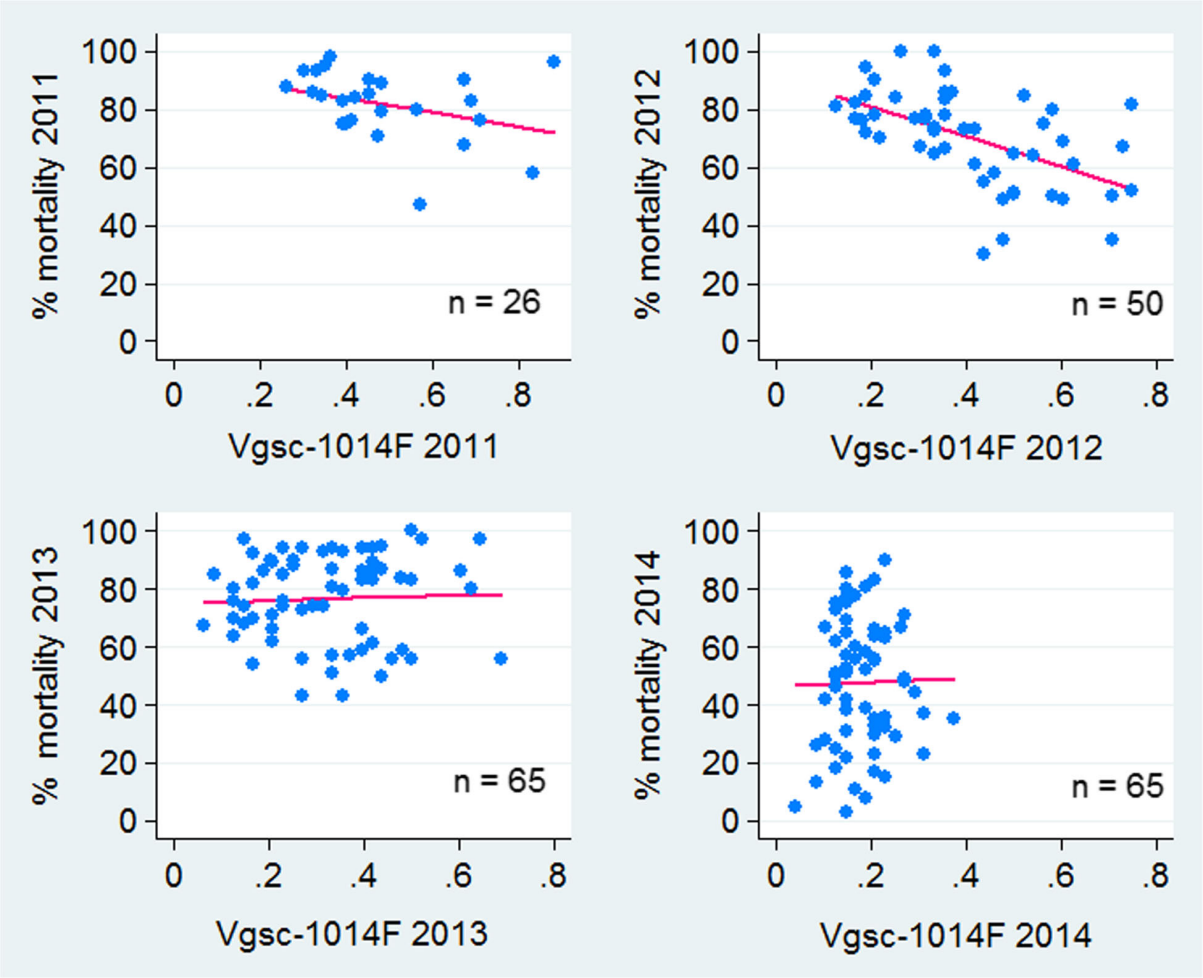
Association between deltamethrin susceptibility (% mortality) and *Vgsc-*1014F frequency for Sudanese *An. arabiensis* populations during 2011–2014

**Fig. 8:**
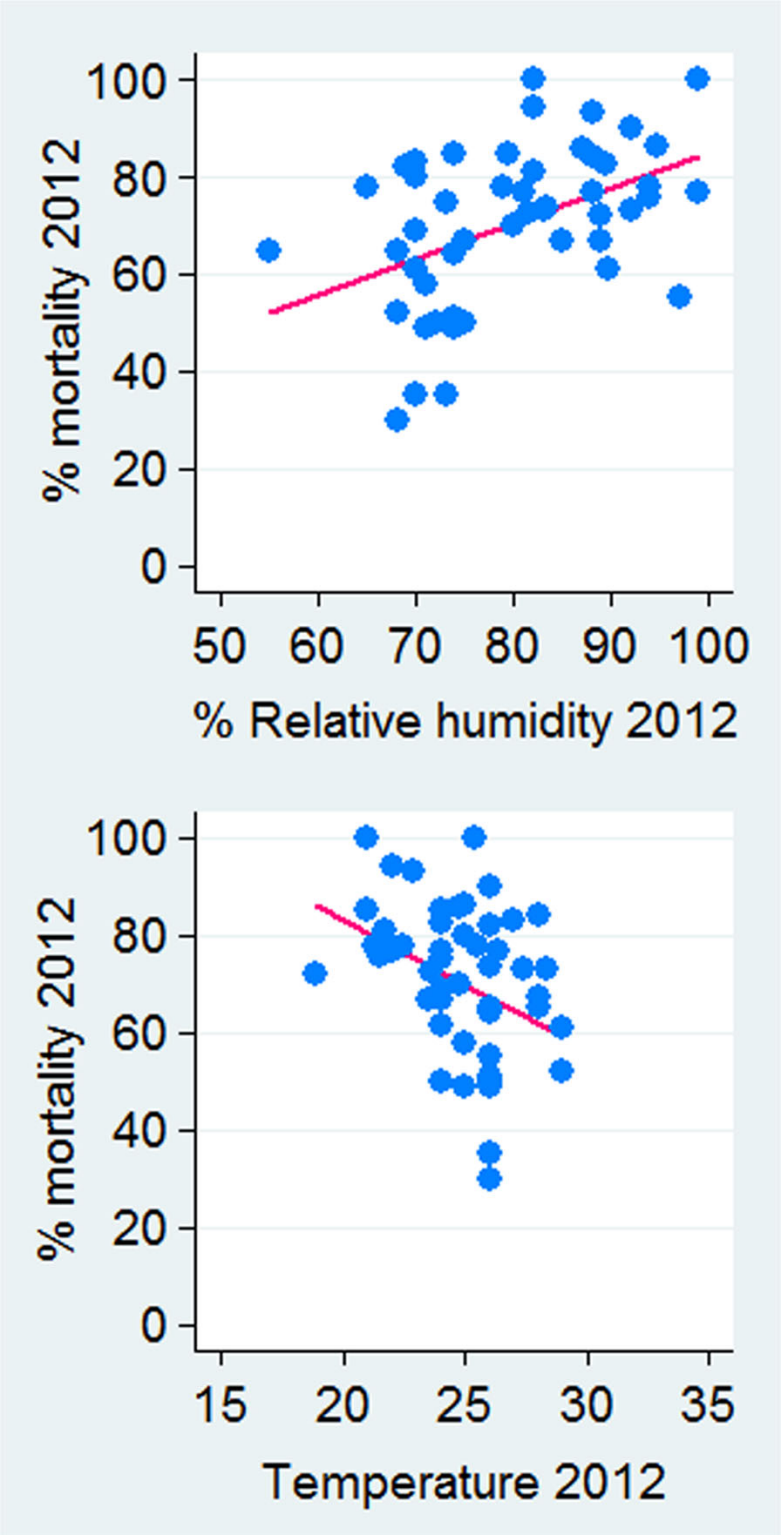
Association between deltamethrin susceptibility % mortality, temperature and relative humidity at 24 h for Sudanese *An. arabiensis* populations in 2012. Humidity (*R*^2^ = 0.21, *P* = 0.009), temperature (*R*^2^ = -0.133, *P* = 0.001)

To determine if *Vgsc-*1014F frequency could be used as a proxy for deltamethrin susceptibility the correlation between *Vgsc*-1014F and deltamethrin mortality was investigated separately (Table 4). A simple mixed effect model account for area and year showed significant association between deltamethrin phenotypic percent mortality and *Vgsc-*1014F (unadjusted coefficient = -47.9, 95% CI: -73.17– -22.66, *P* < 0.0001), temperature (-1.26, CI: -2.56– -0.002, *P <* 0.05) and with relative humidity (*P >* 0.05). The multilevel mixed effect model confirmed the correlation between phenotypic percent mortality and *Vgsc-*1014F (adjusted coefficient = -44.83, 95% CI: -70.73– -18.93, *P <* 0.001). However, the breakdown of the data by year showed a marked significant association between 2012 phenotypic percent mortality and *Vgsc-*1014F [coefficient = - 52.0 % (95% CI: -75.2– -28.7, *P* = 0.001)] (Fig. 7), temperature and humidity (Fig. 8), but, no association was observed for 2011, 2013 and 2014 data sets.

**Table 4:** The effect of *Vgsc-*1014F mutation, temperature and relative humidity on deltamethrin susceptibility % mortality in *An. arabiensis* populations from Sudan 2011–2014

Variable	Unadjusted coeficient (95% CI)	*P*-value	Adjusted coefficient^a^ (95% CI)	*P*-value	Effect size^b^
*Vgsc-*1014F	-47.9 (-73.17, -22.66)	< 0.0001	-44.83 (-70.73, -18.93)	0.001	0.0154
Temperature	-1.26 ( -2.56, -0.002)	0.050	-1.00 (-2.41, -0.407)	0.164	
Relative humidity	-0.063 (-0.143, 0.271)	0.545	-0.019 (-0.252, 0.214)	0.871	

^a^Adjusted for study area and study year

^b^Effect size for mixed random effect model

## Discussion

### Species identification

*Anopheles arabiensis* was the only member of the *An. gambiae* complex that was observed in all 140 clusters from the four study areas. These findings confirmed the previous studies carried out in Gezira and Sennar [2], eastern and northern Sudan [3, 29, 39], and Khartoum State [18] which showed that *An. arabiensis* is the predominant sibling species in these areas. In a cytogenetic study that collected *Anopheles* mosquito specimens from 40 sentinel sites on a country-wide scale, it was revealed that 94% were *An. arabiensis*, while, *An*. *gambiae* (*s*.*s*.) was restricted to the southern part of the country [5].

### Resistance phenotyping

This is the most comprehensive insecticide resistance monitoring programme that has been conducted in Sudan and to our knowledge is the largest known dataset on insecticide resistance. Overall, *An. arabiensis* populations from all sentinel sites across the four study areas were susceptible to bendiocarb. In Sudan, all studies undertaken to date indicated that *An. arabiensis* populations showed no evidence of resistance to bendiocarb insecticide [2, 18, 20]. Similarly, in Uganda and Cameroon, both species *An. arabiensis* and *An. gambiae* (*s.s.*) were found fully susceptible to bendiocarb [40, 41]. In contrast, Ethiopian *An. arabiensis* was found to be susceptible in the South but resistant in the North [42], whilst in Chad, it was susceptible in the North and resistant in the southern parts of the country [43].

Conversely, in the West African continent, resistance to carbamate insecticides was reported in *An. gambiae* (*s.l*.), from Cote d’Ivoire [44], Benin [45], Burkina Faso [46, 47], Mali [48] and South Africa [49]. *Anopheles arabiensis* populations from all sentinel sites exhibited high levels of resistance to deltamethrin and DDT. Previous studies have reported *An. arabiensis* resistant to pyrethoids and DDT in Gezira and Sennar state [2], White Nile [20], Khartoum [50] and in the eastern part of the country [19]. In fact the two study areas of Gezira State (El Hoosh and Hag Abdalla) and New Halfa are located within irrigated schemes in the country, where all classes of insecticides have been used intensively over many years for agricultural purposes. Therefore, the increases in resistance may be due to selection presure from insecticides used in agricultural activities.

Recent studies have suggested that the rapid increase in pyrethroid resistance in *An. gambiae* (*s.s.*) and *An. arabiensis* is correlated with insecticide use for control of cotton and rice pests [44, 51–53]. However for Galabat this may be an unlikely explanaiton as this area is characterized by rain-fed agriculture with only sorghum being cultivated and there is no history of intensive use of agricultural insecticides. It is possible that resistance has emerged in the area through active or/and passive dispersals of resistant *An. arabiensis*, from neighbouring states (i.e. Gezria and Kassala) or bordering countries such as Ethiopia [42, 54].

### Resistance genotyping

Results of this study revealed that the *Vgsc*-1014F resistance mutation was more geographically widespread than previously documented. The *Vgsc-*1014S was detected only in three study areas during 2010 and at very low allele frequencies. However, after the intervention (2011–2014) this mutation was not detected in any *An. arabiensis* population screened. In Sudan *Vgsc-*1014S has previously been reported in population from Kassala (frequency = 0.16) [21] and Khartoum (frequency range = 0.14–0.26) [25] suggesting a marked decline in frequency. Earlier studies from several locations in the country have reported that *An. arabiensis* populations exhibited variable levels of *Vgsc-*1014F frequency [2, 21–23].

Results from the current study show the *Vgsc-*1014F frequency declining over time in all study areas and in both arms. This decline in *Vgsc*-1014F frequency, which has not been reported elsewhere, is in contrast to the increasing levels of phenotypic resistance and suggests that alternative resistance mechanisms are playing a more important role in the study areas. Recent studies have documented that the over-expression of detoxification enzymes in *An. arabiensis* is correlated with pyrethroid resistance [55, 56].

### Impact of combined interventions on resistance evolution

Insecticide resistance management (IRM) has received much attention, and various approaches have been suggested to delay the emergence of resistance [57]. However, all these strategies for malaria vectors have mainly been evaluated and compared using mathematical models [58], and a few experimental hut studies [59–63]. Recently, the WHO has recommended combination interventions with non-pyrethroid insecticides in the same geographical area to delay the emergence of insecticide resistance [57]. We feel that the most important finding from the present study is that combining bendiocarb IRS with LLINs slowed down the speed of selection for pyrethroid insecticide. This was a replication of an earlier analysis of data from Galabat which showed a similar retardation of the speed of resistance evolution [28]. A recent cluster randomized trial of LLIN *vs* LLIN + IRS (carbamate) in Tanzania showed a broadly similar pattern [64].

### Genotype phenotype associations

Results from the current study (2012) demonstrate a significant negative association between genotypic and deltamethrin phenotypic mortality at the population level, but not for the three other years of data. There are numerous studies showing that in *An. arabiensis kdr* (L1014F) is a strong predictor of pyrethroid resistance at the individual level [65–68]. However, relative humidity and temperature under which bioassay tests for phenotypic resistance were conducted were shown to have a major impact on mortality outcomes and should be carefully documented and controlled.

## Conclusions

The present study documented that deltamethrin and DDT resistance is geographically widespread in Sudanese *An. arabiensis*. Importantly for efforts to delay resistance emergence we provide valuable proof-of-principle data that demonstrates the possibility of delaying pyrethroid phenotypic resistance development by combining pyrethroid-LLINs with a non-pyrethroid IRS (bendiocarb) compared to LLIN alone.

## Additional files

Additional file 1: **Table S1.** Mean % mortality (95% CI) of female *An. arabiensis* populations from the four study areas exposed to standard WHO discriminating concentration of bendiocarb in Sudan 2011–2014. (DOC 37 kb)

Additional file 2: **Table S2.** Mean % mortality (95% CI) of *An. arabiensis* populations from four study areas exposed to standard WHO discriminating concentration of DDT in Sudan 2011–2014. (DOC 39 kb)

Additional file 3: **Table S3.** Mean % mortality (95% CI) of *An. arabiensis* populations from four study areas exposed to standard WHO discriminating concentration of deltamethrin in Sudan 2011–2014. (DOC 37 kb)
