# Supplementary material for: Temporal and spatial trends in insecticide resistance in Anopheles arabiensis in Sudan: outcomes from an evaluation of implications of insecticide resistance for malaria vector control
Source: Parasit Vectors. 2018 Mar 2;11:122. doi: 10.1186/s13071-018-2732-9 (PMC5834846; doi:10.1186/s13071-018-2732-9)
Supplement: Supplementary file 1 — Table S1. Mean % mortality (95% CI) of female An. arabiensis populations from the four study areas exposed to standard WHO discriminating concentration of bendiocarb in Sudan 2011–2014. (DOC 37 kb) [file 13071_2018_2732_MOESM1_ESM.doc]

Table S1. Mean % mortality (95% C.I.) of *An. arabiensis* populations from the four study areas exposed to standard WHO discriminating concentration of bendiocarb in Sudan 2011-2014.

| Area  Year | *n* | El Hoosh | *n* | Hag Abdalla | *n* | Galabat | *n* | New Halfa | *n* | Overall % mean**^a^**  [95% C.I.] |
| --- | --- | --- | --- | --- | --- | --- | --- | --- | --- | --- |
| 2011 | 13 | 99.3a  [97.1 - 100] | 11 | 98.8%a  [95 - 100] | 8 | 99.2%a  [98.3 - 100] | 7 | 97%a  [96.2 - 97.8] | 39 | 98% A  [97.5 - 99.9] |
| 2012 | 14 | 96.1a  [94 - 98.2] | 16 | 93.9%a  [90.8 - 97.1] | 7 | 97.5%a  [96.4 - 98.5] | 5 | 98.7%a  [97.8 - 99.7] | 42 | 95.8% B  [94.6 - 97] |
| 2013 | 14 | 97.8a  [95.7 - 99.9] | 15 | 99.4%a  [96.1 - 100] | 10 | 99.9%a  [99 - 100] | 14 | 100%a  [99.4 -100] | 53 | 99.2% A  [98.2 - 100] |
| 2014 | 10 | 100a  [97.5 - 100] | 10 | 99.4%a  [95.3 - 100] | 9 | 100%a  [99 - 100] | 16 | 99.8%a  [99.3 - 100] | 45 | 99.8% A  [98.7 - 100] |
| Overall % mean**^b^**  [95% C.I.] | 51 | 98.1%A  [97.0 - 99.3] | 52 | 97.6%A  [96.4 - 98.7] | 34 | 99.2%A  [98.0 - 99.9] | 42 | 99.3%A  [98.0 - 100] |  |  |

*Abbreviation*: n, number of sentinel clusters for which mortality data are available; Colum’s not sharing the same lowercase letter are significantly different (p<0.05).

**^a,b^**; Overall mean colum or raw not sharing the same UPPERCASE letter are significantly different (p<0.05).
