# Supplementary material for: Temporal and spatial trends in insecticide resistance in Anopheles arabiensis in Sudan: outcomes from an evaluation of implications of insecticide resistance for malaria vector control
Source: Parasit Vectors. 2018 Mar 2;11:122. doi: 10.1186/s13071-018-2732-9 (PMC5834846; doi:10.1186/s13071-018-2732-9)
Supplement: Supplementary file 2 — Table S2. Mean % mortality (95% CI) of An. arabiensis populations from four study areas exposed to standard WHO discriminating concentration of DDT in Sudan 2011–2014. (DOC 39 kb) [file 13071_2018_2732_MOESM2_ESM.doc]

Table S2. Mean % mortality (95% C.I.) of *An. arabiensis* populations from the four study areas exposed to standard WHO discriminating concentration of DDT in Sudan 2011-2014

| Area  Year | *n* | El Hoosh | *n* | Hag Abdalla | *n* | Galabat | *n* | New Halfa | *n* | Overall % mean**^a^**  [95% C.I.] |
| --- | --- | --- | --- | --- | --- | --- | --- | --- | --- | --- |
| 2011 | 9 | 57.3% a  [48.1- 66.5] | 5 | 69.7%a  [55.1 - 84.3] | 4 | 51.7%a  [44.1- 59.3] | 4 | 56.8%a  [41.6 - 72.0] | 22 | 59.1%A  [52.2 - 65.9] |
| 2012 | 7 | 76.7%b  [66.2 - 87.1] | 9 | 83.6%ab  [72.7 - 94.5] | 2 | 60%a  [49.3 - 70.6] | 11 | 64.1%a  [55.0 - 73.3] | 29 | 72.9%B  [67.0 - 78.9] |
| 2013 | 12 | 66.2%a  [58.2 - 74.1] | 13 | 69.4%ab  [60.3 - 78.5] | 8 | 95.5%b  [90.1- 100] | 14 | 61.3%a  [53.1- 69.4] | 47 | 70.6%B  [65.9 - 75.3] |
| 2014 | 15 | 65.8%a  [58.6 - 72.9] | 15 | 63.9%ac  [55.4 - 72.3] | 1 | 85%b  [NA] | 12 | 61.7%a  [52.9 - 70.5] | 43 | 66.4%aB  [61.7 - 71.0] |
| Overall % mean**^b^**  [95% C.I.] | 43 | 65.9%A  [61.1 - 70.7] | 42 | 70.5%A  [65.6 - 75.4] | 15 | 78.4%AB  [70.2 - 86.5] | 41 | 61.7%AC  [56.8 - 66.7] |  |  |

*Abbreviation*: n, number of sentinel clusters for which mortality data are available; Colum’s not sharing the same lowercase letter are significantly different (p<0.05).

**^a,b^**; Overall mean colum or raw not sharing the same UPPERCASE letter are significantly different (p<0.05).

NA; not applicable
