# Supplementary material for: Temporal and spatial trends in insecticide resistance in Anopheles arabiensis in Sudan: outcomes from an evaluation of implications of insecticide resistance for malaria vector control
Source: Parasit Vectors. 2018 Mar 2;11:122. doi: 10.1186/s13071-018-2732-9 (PMC5834846; doi:10.1186/s13071-018-2732-9)
Supplement: Supplementary file 3 — Table S3. Mean % mortality (95% CI) of An. arabiensis populations from four study areas exposed to standard WHO discriminating concentration of deltamethrin in Sudan 2011–2014. (DOC 37 kb) [file 13071_2018_2732_MOESM3_ESM.doc]

Table S3. Mean % mortality (95% C.I.) of *An. arabiensis* populations from the four study areas exposed to standard WHO discriminating concentration of deltamethrin in Sudan 2011-2014

| Area  Year | *n* | El Hoosh | *n* | Hag Abdalla | *n* | Galabat | *n* | New Halfa | *n* | Overall % mean**^a^**  (95% C.I.) |
| --- | --- | --- | --- | --- | --- | --- | --- | --- | --- | --- |
| 2011 | 14 | 84.9%a  [77.2 - 92.7] | 15 | 86.2%a  [78.8 - 93.6] | 11 | 79.0%a  [70.5 - 87.6] | 9 | 68.6%a  [56.9 - 80.4] | 49 | 81.0%A  [76.2 - 85.8] |
| 2012 | 15 | 78.9%a  [71.5 - 86.6] | 13 | 78.6%a  [70.7 - 86.5] | 11 | 62.8%ab  [54.2 - 71.3] | 11 | 55.9%a  [45.2 - 66.5] | 50 | 70.2%B  [65.5 - 74.9] |
| 2013 | 18 | 73.5%a  [66.6 - 80.3] | 18 | 77.7%a  [70.9 - 84.4] | 12 | 86.7%ac  [78.5 - 94.9] | 17 | 72.0%a  [63.4 - 80.5] | 65 | 76.7%AB  [72.5 - 80.8] |
| 2014 | 18 | 49.5%b  [42.6 - 56.3] | 18 | 48.0%b  [41.3 - 54.8] | 11 | 67.3%ab  [58.7 - 75.8] | 18 | 33.4%b  [25.1 - 41.7] | 65 | 47.7%C  [43.5 - 51.8] |
| Overall % mean**^b^**  (95% C.I.] | 65 | 70.5%A  [65.6 - 75.5] | 64 | 71.5%A  [66.5 - 76.5] | 45 | 74.2%A  [68.2 - 80.2] | 55 | 55.6%B  [50.2 - 61.1] |  |  |

*Abbreviation*: n, number of sentinel clusters for which mortality data are available; Colum’s not sharing the same lowercase letter are significantly different (p<0.05).

**^a,b^**; Overall mean colum or raw not sharing the same UPPERCASE letter are significantly different (p<0.05).
